# Reliability and validity of CDAI and SDAI indices in comparison to DAS-28 index in Moroccan patients with rheumatoid arthritis

**DOI:** 10.1186/s12891-015-0718-8

**Published:** 2015-09-29

**Authors:** Imane Ben Slama, Fadoua Allali, Touria Lakhdar, Sarra El kabbaj, Lamyae Medrare, Ange Ngeuleu, Hanan Rkain, Najia Hajjaj - Hassouni

**Affiliations:** Department of Rheumatology, El Ayachi Sale University-Hospital, Sale, 11000 Morocco; Laboratory of Information and Research on Bone Diseases (LIRPOS-URAC 30), Mohamed V University, Souissi Rabat, Rabat 10000 Morocco; Biostatistics Laboratory, Clinical Research and Epidemiology (LBRCE), Faculty of Medicine and Pharmacy, Mohammed V University, Souissi Rabat, Rabat 10000 Morocco; Laboratory of Physiology, Faculty of Medicine and Pharmacy, Mohamed V University, Souissi Rabat, Rabat 10000 Morocco

**Keywords:** Clinical disease activity index, Simplified disease activity index, Disease activity score 28 joints, Rheumatoid arthritis activity

## Abstract

**Background:**

Clinical disease activity index (CDAI) and simplified disease activity index (SDAI) are useful tools for the evaluation of disease activity in patients with rheumatoid arthritis (RA), but have not been comparatively validated in Moroccan population. Therefore, this study was designed to assess validity and reliability of CDAI and SDAI in comparison to disease activity score-28 joints (DAS-28) in Moroccan patients with RA.

**Methods:**

Patients with RA were included in a cross-sectional study. Patient characteristics and RA were collected. The disease activity was assessed by DAS-28, CDAI and SDAI. Patients were splitted into groups of remission, low, moderate and high activity on the basis of predefined cut-offs for DAS-28, CDAI, and SDAI. A Spearman correlation between composite indexes and inter-group comparison of the indexes were performed. Using DAS-28 as a gold standard, the Receiver operator characteristic (ROC) curve was used to assess the performance of a screening test at different levels.

**Results:**

The study was conducted with 103 patients of female predominance (87.4 %). Mean age was 49.7 ± 11.4 years. Median disease duration was in the order of 8 years [3-14]. There was an excellent correlation between DAS-28 and CDAI (*r* = 0.95, *p* <0.001), CDAI and SDAI (*r* = 0.90, *p* <0.001), and DAS-28 and SDAI (*r* = 0.92, *p* <0.001). There was a good inter-rater alignment between the DAS-28 and CDAI (Weighted kappa =0.743) and there was a moderate inter-rater alignment between the DAS-28 and SDAI (Weighted kappa =0.60), and also between the SDAI and CDAI (Weighted kappa = 0.589). There was no statistically significant difference between AUROC of CDAI and SDAI as both were performed equally well.

**Discussion:**

This study is the first Moroccan case study to compare the performance of both CDAI and SDAI in evaluation of disease activity in patients with RA. Our study showed that there was a direct and excellent correlation between DAS-28 and CDAI, and SDAI and DAS-28.

**Conclusion:**

Our study shows a strong positive correlation between DAS-28, CDAI and SDAI. The cut-off values for CDAI and SDAI used in western literature can be used with minor modifications in Moroccan scenario.

## Background

Rheumatoid arthritis (RA) is a systemic autoimmune disease whose main characteristic is persistent joint inflammation that results in joint damage and loss of functions. These adverse consequences can be prevented, at least partially, by early appropriate therapy, particularly a “tight control” strategy [1]. Such strategy requires evaluating disease activity and reaction to treatment using objective and standardized tools [2].

The currently available disease composite activity indexes that provide a single number on a continuous scale are the Disease Activity Score (DAS), the DAS using 28 joint counts (DAS- 28) [3], the Simplified Disease Activity Index (SDAI) [3], and the Clinical Disease Activity Index (CDAI) [3].

Until recently DAS-28 was the only gold standard to measure the disease activity in patients with RA [4]. It is recommended by the European League Against Rheumatism (EULAR) [5]. DAS28 is calculated from the number of tender and swollen joints (28-joint count), patient self-assessment of disease activity (visual analog scale), and ESR by the following formula: DAS28 = (0.56 * tender joint count 1/2) + (0.28 * swollen joint count 1/2) + (0.7 * ln [ESR]) + (0.014*VAS) [6]. This means that this formula requires very complicated calculation and therefore a calculator is needed. So it is often difficult to do it practically on a daily basis for patients consultation.

Clinical Disease Activity Index (CDAI) is a composite index (without acute-phase reactant) for assessing disease activity. CDAI is based on the simple summation of the count of swollen/tender joint count of 28 joints along with patient and physician global assessment on VAS (0–10 cm) Scale for estimating disease activity [3]. The greatest advantage of CDAI is the omission of laboratory test. Therefore, it can essentially be used everywhere and at anytime for disease activity assessment on RA patients [7].

The simplified disease activity index (SDAI) is a quick and convenient method for measuring rheumatoid arthritis in a clinical environment. It is scored by simply adding the numerical values corresponding to the following set of predetermined elements: the 28 joint assessment used to measure tender and swollen joint count; patient and physician global assessment of disease activity measured using a visual analogue scale, and finally, levels of the measured C-reactive protein (mgudl, normal <1 mgudl) [8].

The SDAI and CDAI were validated in the original studies that were developed in using additional cohorts of patients [9]. Therefore, the aim of the present study was to assess disease activity in Moroccan patients with RA using CDAI and SDAI and to evaluate reliability and validity of CDAI and SDAI in comparison to DAS-28 in Moroccan patients with rheumatoid arthritis.

## Methods

A total of 103 RA cases were included in a cross-sectional study in the Department of Rheumatology, at El Ayachi hospital in Morocco, The period of data collection was from October 2012 to March 2013. Patients were diagnosed to have RA by the rheumatologist according to American College of Rheumatology (ACR 1987) classification Criteria for RA [10]. Patients with diseases other than rheumatoid arthritis were excluded from the study. Our study is non-interventional and verbal consent was obtained from all the patients. The study was approved by ethics committee of our university hospital (El Ayachi University-Hospital Sale, Morocco).

Disease history, clinical examination, and routine laboratory investigations including radiographical examination were all detailed for the subjects included in the study. All patients were asked about their age, duration of the disease, visual analogue scale of pain (0–100 mm), the morning stiffness in minutes, the number of swollen joints (0–28) and tender joints (0–28), medication taken and Erythrocyte sedimentation rate (ESR). The disease activity was assessed by DAS-28 ESR, CDAI and SDAI.

Patients were splitted into groups of remission, low, moderate and high activity on the basis of predefined cut-offs for DAS-28, CDAI, and SDAI [9].

Statistical analysis: Statistical analysis was done using statistical package for social sciences version 13 (SPSS 13.0) and MedCalc statistical software.

Spearman correlation coefficient (r) was used to assess the correlation between continuous variables of indexes (DAS-28, CDAI and SDAI). Weighted Kappa statistics (Weighted K) were used to assess the alignment between each score. We used Altman 1991 guidelines for kappa grading (<0.20 as poor, 0.21–0.40 as fair, 0.41–0.60 as moderate, 0.61–0.80 as good, and 0.81–1.00 as very good) [11]. Findings with P value less than 0.05 were considered significant.

Using DAS-28ESR as gold standard, the sensitivity and specificity of CDAI and SDAI cut offs were determined to predict levels of disease activity by area under receiver operator characteristics curves (AUROC).

## Results

Of the 103 patients who were included in the study, 90 were females and 13 were males. The mean age of the patients was 49.7 ± 11.4 years (mean ± SD) and the median duration of illness was 8 (3–14) years. Demographic profile of patients and the mean values by core set of variables were shown in (Table 1).

**Table 1:** Demographic and clinical characteristics of RA patients (*n* = 103)

Characteristics	N = 103
Age (Years mean ± SD)	49,7 ± 11,4
Sex (female/male)	90/13
Median duration of illness (per years)	8 (3–14)^a^
VAS (mean ± SD) (mm)	40 ± 29
HAQ	0,5 (0–1,37)^a^
DAS 28 ESR	4,27 ± 1,75
CDAI	13 (5,25)^a^
SDAI	24 (11,40)^a^

*VAS* Visual analogue scale of pain, *HAQ* Health assessment questionnaire, *DAS28* Disease activity for 28 joint indices score, *ESR* Erythrocyte sedimentation rate, *CDAI* Clinical disease activity index, *SDAI* Simplified disease activity index

^a^median and quartiles (the 50th percentile)

Patients were put under 4 groups of disease activity based on predefined cut off values of DAS-28, CDAI and SDAI (Table 2). Most of our patients (›60 %) were classified under moderate and high disease activity when DAS-28, CDAI and SDAI criteria were used.

**Table 2:** Distribution of patients with various levels of disease activity and criteria used in our study

Index	Disease of activity	Definition	Number of patients
DAS-28 ESR	Remission	<2.6	20
	Low activity	<3.2	20
	Moderate activity	<5.1	42
	High activity	≥5.1	21
CDAI	Remission	<2.8	16
	Low activity	<10	25
	Moderate activity	<22	31
	High activity	≥22	31
SDAI	Remission	<3.3	5
	Low activity	<11	19
	Moderate activity	<26	24
	High activity	≥26	49

*DAS28* Disease activity for 28 joint indices score, *ESR* Erythrocyte sedimentation rate, *CDAI* Clinical disease activity index, *SDAI* Simplified disease activity index

**Fig. 1:**
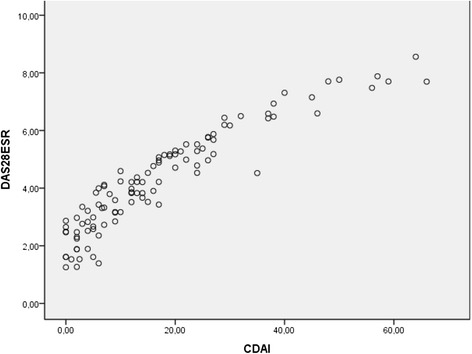
Scatter diagram showing linear positive correlation between CDAI and DAS-28 scores (Spearman’s correlation coefficient (rho) = 0.95, *p* <0.001

**Fig. 2:**
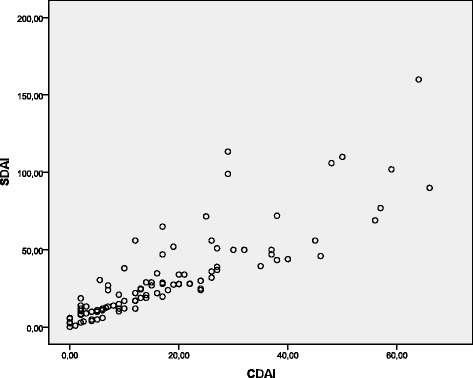
Scatter diagram showing linear positive correlation between SDAI and CDAI (Spearman’s correlation coefficient (rho) = 0.90, *p* <0.001

**Fig. 3:**
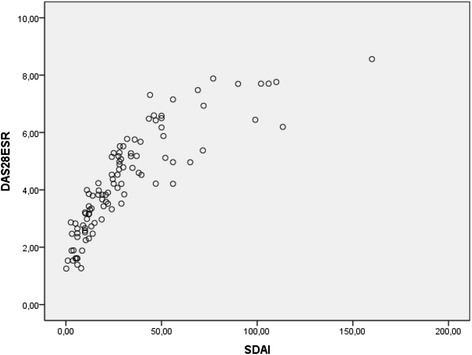
Scatter diagram showing linear positive correlation between SDAI and DAS-28 scores (Spearman’s correlation coefficient (rho) = 0.92, *p* <0.001

There was an excellent correlation between DAS-28 and CDAI (*r* = 0.95, *p* <0.001) (Fig. 1), CDAI and SDAI (*r* = 0.90, *p* <0.001) (Fig. 2), and DAS-28 and SDAI (*r* = 0.92, *p* <0.001) (Fig. 3).

There was a good inter-rater alignment between the DAS-28 and CDAI (weighted k = 0.743) and there was a moderate inter-rater alignment between the DAS-28 and SDAI (weighted k = 0.60), and between the SDAI and CDAI (weighted k = 0.589) (Table 3).

**Table 3:** Measurement of agreement between DAS28, CDAI and SDAI in 103 RA patients

Variables	Weighted K value	*P* value
CDAI	0.743	<0.001
SDAI	0.60	<0.001

**Fig. 4:**
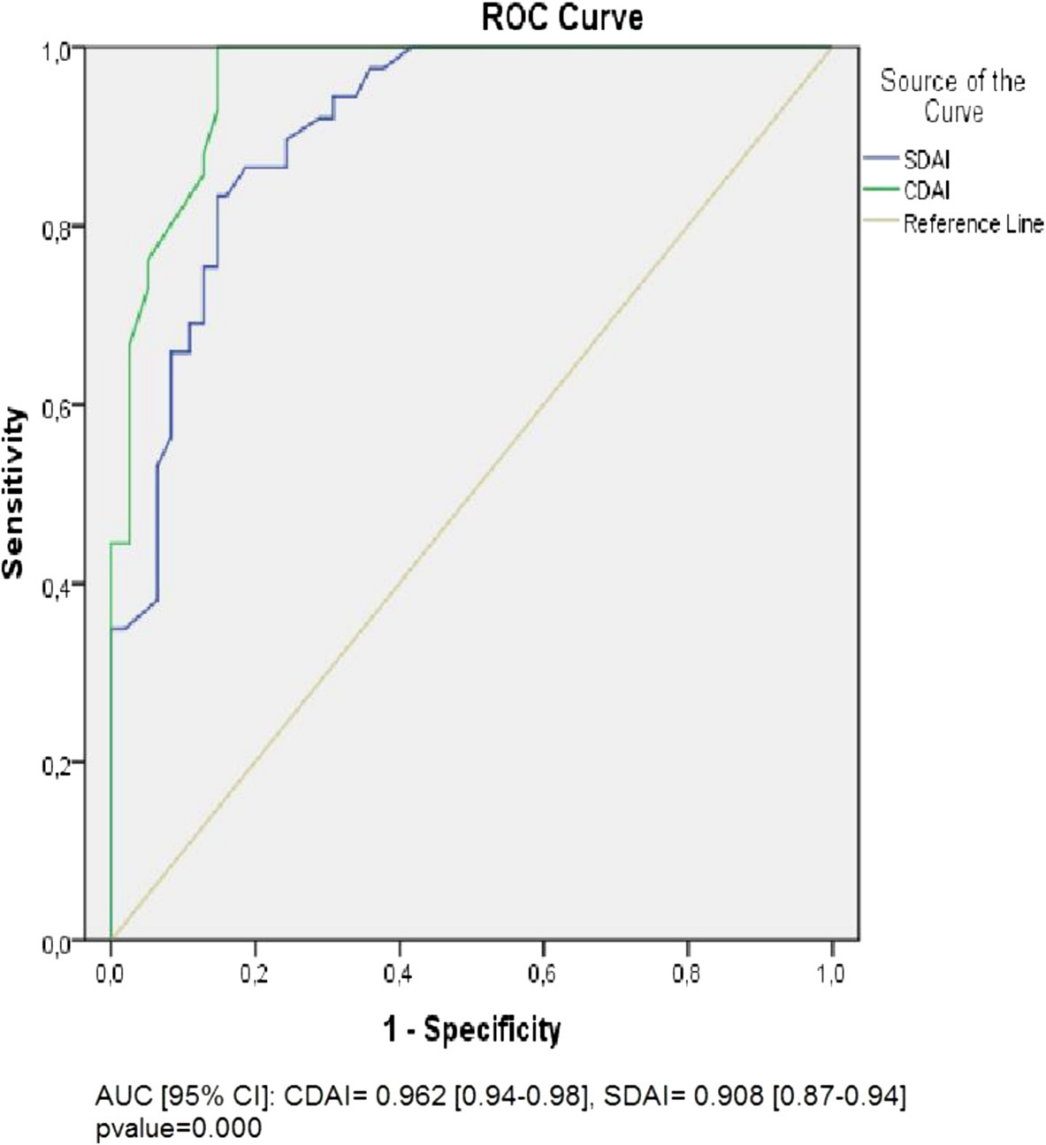
ROC curve illustrating the sensitivity and 1-specificity values for different values of CDAI corresponding to a DAS28>5.1; AUC, area under the curve; CI, confidence interval

A Receiver Operating Characteristic (ROC) curve (Fig. 4) was constructed to determine the sensitivity and specificity of different values of CDAI and SDAI which would differentiate between a DAS28 value greater than and less than 5.1 (high disease activity). The best combination of sensitivity (97 %) and specificity (85.3 %) was provided by a CDAI value of 18.5 (with 95 % confidence interval 94.2–98.1). Similarly the highest sensitivity (97.6 %) and specificity (62.2 %) were given by SDAI value of 24 (with 95 % confidence interval 87.5–94.18) (Table 4).

**Table 4:** Validity of CDAI and SDAI in comparison to DAS28 in 103 RA patients

Test variable	AUC	95 % CI	Sensitivity	Specificity	*p* value
CDAI	0.962	0.94–0.98	97 %	85.3 %	0.000
SDAI	0.908	0.87–0.94	97.6 %	62.2 %	0.000

## Discussion

This study is the first Moroccan case study to compare the performance of both CDAI and SDAI in evaluation of disease activity in patients with RA.

Our study showed that there was a direct and excellent correlation between DAS-28 and CDAI, and SDAI and DAS-28. The comparison of the number of patients under each disease activity category, according to the disease activity indexes using weighted kappa-statistics, revealed a good alignment between the CDAI and DAS-28 but a moderate alignment between the SDAI and DAS-28. The excellent correlation was only at group level but not per each single patient. In addition to that, CDAI had high sensitivity, high specificity and high area under the curve. These results agreed with other studies which showed that CDAI is a valid and comparable tool to DAS28 [12–14]. We found no significant difference in test performance and AUROC of CDAI and SDAI while assessing disease activity. This suggests that CDAI is a valid alternative to SDAI as seen in many studies [3, 15].

We also observed that the cut offs suggested by our study and other various Western studies vary slightly and hence the proposed EULAR cut offs can be used universally to differentiate between different grades of disease activity with only minor modifications. These results were also found in Indian population [15].

The management of RA has changed radically over the last 10 years, with the introduction of new drugs and treatment strategies and with the emergence of new concepts of disease severity, treatment targets, and means of evaluating treatment effects [13]. The CDAI is a more simplified than the DAS28 because it is a simple summation score requiring nothing more complex than addition [9]. Furthermore, our study showed that CDAI performs equally well as SDAI. So we suggest that this simple clinical tool should be used more often instead of ordering ESR/CRP at every patient visit and without the need of any calculating device. Since it can essentially be evaluated everywhere and at anytime, it may facilitate decision-making by physicians and helps to avoid lags in efficient treatment adaptation for RA patients. Such scores may be easier to understand by the patients and encourage them to keep track of their “index”. This can improve patient’s adherence to treatment regimen.

The strength of this study resides in comparing the performance of both CDAI and SDAI for measuring disease activity and deriving sensitivity and specificity for cut offs proposed by various studies, which has not been done previously in any Moroccan study.

The small number of patients included in our study may be seen as a limited sample. Therefore, other studies with a larger patient number would be considered more interesting.

## Conclusion

On the basis of the study results and related statistics, it is suggested that CDAI and SDAI had good correlation with DAS-28 for disease activity assessment in Rheumatoid Arthritis patients. The cut-off values for CDAI and SDAI used in western literature can be used with minor modifications in Moroccan scenario. In contrast to DAS28 and SDAI, CDAI can be obtained at any time and in any setting without the need of any lab value or any calculator/computer device. Therefore, CDAI is a very useful disease activity assessment tool in daily clinical practice for RA patients.
